# Sequence modeling tools to decode the biosynthetic diversity of the human microbiome

**DOI:** 10.1128/msystems.00333-25

**Published:** 2025-06-30

**Authors:** Mohammed Salim Dason, Davide Corà, Angela Re

**Affiliations:** 1 Department of Applied Science and Technology (DISAT), Politecnico di Torino, Torino, Italy; 2 Department of Translational Medicine (DIMET), University of Piemonte Orientale, Novara, Italy; 3 Center for Translational Research on Autoimmune and Allergic Disease (CAAD), Novara, Italy; Medizinische Universitat Graz, Graz, Austria

**Keywords:** biosynthetic gene cluster, genome mining, self-supervised learning, hidden Markov model, graph analysis, natural product discovery

## Abstract

Understanding the biosynthetic potential of the human microbiome remains a significant challenge with far-reaching scientific and translational implications. Analyses of human-associated (meta)genomic sequencing data undeniably show that the biosynthetic diversity encoded in these genomes is largely underexplored. A crucial step in studying specialized metabolites involves the sequence-based identification of genes encoding biosynthetic pathways, typically organized into biosynthetic gene clusters (BGCs). In this review, we provide a concise and updated overview of the widening range of computational approaches that have effectively addressed the sequence-based identification of BGCs across both isolated genomes and complex microbial communities. These advancements are set to deepen our understanding of the biosynthetic potential and diversity of microorganisms residing in different human body sites.

## INTRODUCTION

The human-associated microbiome is a complex and dynamic consortium of microorganisms residing in and on the human body, fulfilling several crucial roles in maintaining health (1–3). Changes in its functional capacity have been implicated in various pathological states, encompassing metabolic disorders, autoimmune diseases, and neurodegenerative conditions (4, 5). The human-associated microbiome harbors remarkable biosynthetic capabilities, producing a wide array of bioactive molecules, including vitamins, short-chain fatty acids, secondary metabolites, and signaling molecules (6). Among these, secondary metabolites—encoded by the genetic repertoire of human-associated microbial ecosystems (7)—have become a focal point for their therapeutic potential and diverse biological activities.

Advances in sequencing technologies, such as activity-guided genomics, amplicon sequencing, and shotgun metagenomics, have significantly improved our ability to investigate the biosynthetic capabilities of the human microbiome (8), and large-scale initiatives, such as the Human Microbiome Project (HMP), have further accelerated this process (9).

**Fig 1:**
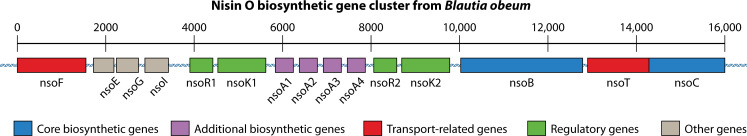
Example of a biosynthetic gene cluster (BGC) encoding Nisin O in *Blautia obeum*. A BGC consists of two or more genes physically clustered on the chromosome of a certain genome, collectively encoding a biosynthetic pathway responsible for the production of a specialized metabolite.

What we have learned from these early studies is that specialized metabolic pathways are often encoded within biosynthetic gene clusters (BGCs), which are distinct genomic loci consisting of two or more co-localized and functionally interconnected genes (Fig. 1) (10). Thus, the systematic identification and functional characterization of these BGCs is set to enhance our understanding of human genetics and biochemistry, leading to the development of new preventive strategies, diagnostic tools, and therapeutics, such as antimicrobials and immunomodulatory agents (8, 11, 12).

The critical role of BGCs is further underscored by their differential representation in health- vs. disease-associated microbiomes (13). Recent progress in annotating gene clusters derived from metagenomic data sets (Table 1) has led to the creation of specialized databases cataloging thousands of human-associated BGCs. Notable examples include (i) the Atlas of Biosynthetic gene Clusters in the Human Microbiome (ABC-HuMi) (14), an interactive platform that facilitates navigation through gene clusters inferred using robust genome mining tools and metagenomic data across five distinct human body sites; and (ii) the Atlas of Secondary Metabolite Biosynthetic Gene Clusters from the Human Gut Microbiome (sBGC-hm) (15), a specialized resource that catalog BGCs exclusively derived from the human gut. An overview of these and other generalist and human-associated BGC databases is available in Table 2, which underscores how the diversity among BGC classes and their associated natural products mirrors the substantial taxonomic variability inherent to the human microbiome across different physiological and pathological conditions (16, 17). Intriguingly, a growing body of evidence indicates that a significant number of identified BGCs remains functionally uncharacterized (12, 13).

**TABLE 1:** Overview of tools for both class-specific and broad-spectrum BGC detection^*a*^

Class	Software tool	Purpose	Algorithmic features	Reference
Generalist	BGCFlow	BGC identification; BGC organization and visualization	Snakemake-based multi-functional BGC analyzer including in-house and third-party analytics for data selection (GTDB-Tk, MASH or fastANI-based genomic distance), functional annotation (Prodigal, eggNOG-mapper), phylogenomic placement (autoMLST), BGC genome mining (antiSMASH, GECCO, and ARTS2), BGC comparative analysis (BiG-SLICE, BiG-SCAPE, BiG-FAM, and Roary)	18
Generalist	IsaBGC (Lineage-specific analysis of BGCs	BGC genome identification; BGC clustering; gene cluster family (GCF) exploration; evolutionary and population genetic analysis of biosynthetic genes	Analytical workflow including in-house and third-party analytics such as Prodigal, KOfam (a customized Hidden Markov Models [HMM] database of KEGG Orthologs) and PGAP (NCBI Prokaryotic Genome Annotation Pipeline) HMMs for gene calling and annotation, antiSMASH, GECCO, and DeepBGC for BGC identification, OrthoFinder2 for phylogenetic orthology inference, Markov chain clustering for BGC clustering into GCFs, custom HMM-based algorithm for GCF identification, GToTree for genome-level evolutionary inference, and gene-based metagenomic analysis	19
Generalist	ClusterFinder	BGC identification	Two-state HMM-based probabilistic algorithm (with hidden states representing BGC and non-BGC)	20
Generalist	antiSMASH (antibiotics and Secondary Metabolite Analysis SHell) 7.0	BGC identification	Two-state HMM-based probabilistic algorithm; BCG class-specific functionalities: trans-AT PKSs-specific profile HMMs; NRPS adenylation domain substrate specificity prediction by NRPyS; CompaRiPPson analysis for novelty assessment in ribosomally synthesized and post-translationally modified peptide (RiPP) precursor peptides	21
Generalist	GECCO (GEne Cluster prediction with COnditional random fields)	BGC identification	Conditional random forests including a feature selection approach based on the two-sided Fisher’s exact test to identify domains associated with BGC presence/absence	22
Generalist	Deep-BGCpred	BGC identification	Stacked bidirectional long short-term memory (Bi-LSTM) neural network for biosynthetic genes prediction combined with random forest multi-label classifier for false positive reduction and BGC class assignment	23
Generalist	BiGCARP (Biosynthetic Gene Convolutional Autoencoding Representations of Proteins)	BGC identification	Embedding of Pfam domain sequences with the ESM-1b protein masked language model; BiGCARP architecture consists of a dilated 1D-convolutional neural network masked language model based on ByteNet and CARP	24
Generalist	TaxiBGC (Taxonomy-guided Identification of Biosynthetic Gene Clusters)	BGC identification	MetaPhlAn3-based species-level taxonomic profiling of shotgun metagenomic sequence data; Minimum Information about the Biosynthetic Gene Cluster (MiBiG) query with identified species; sequence aligner for MiBiG BGC confirmation in metagenomic sequences	25
Generalist	DeepBGC	BGC identification; NP^*b*^ classification	Bi-LSTM recurrent neural network; word2vec-like word embedding skip-gram neural network (pfam2vec); random forest multi-label classifier for BGC product class prediction	26
Generalist	PRISM (PRediction Informatics for Secondary Metabolomes)	BGC identification; prediction of NP structure	Profile HMMs for enzymatic domains identification; greedy algorithm for BGC identification; combinatorial linear scaffold-based approach for NP structure prediction; BGC/NP dereplication	27
Generalist	PRISM 3	BGC identification; prediction of NP structure	Profile HMMs for enzymatic domains identification; chemical graph-based approach for structure prediction	28
Generalist	PRISM 4	BGC identification; prediction of NP structure	Profile HMMs, conserved protein motifs, and machine-learning classifiers for enzymatic domains identification; combinatorial chemical graph-based approach for structure prediction	29
Generalist	NPOmix (Natural Products Mixed Omics)	MS-guided BGC identification	K-nearest neighbor algorithm based on similarity BGC fingerprints and similarity MS/MS fingerprints to classify GCFs for each MS/MS spectrum; antiSMASH and BiG-SCAPE for BGC and GCF discovery; GNPS-based cosine score for MS/MS spectra similarity computation	30
Generalist	NPLinker	MS-guided BGC/GCF identification	BGCs clustering into GCFs by BiG-SCAPE; GNPS-based spectral clustering of MS/MS spectra into molecular families (MFs); feature-based approach to link BGCs to MS/MS spectra via input-output kernel regression or correlation-based approach to link GCFs to MFs or MS/MS spectra or the combination of correlation- and feature-based approaches	31
Class specific	TrRiPP	RiPP BGC identification	Transformer encoder generating the input of two layers of Bi-LSTM; concatenated maximum, mean, and last outputs from the last Bi-LSTM layer are subjected to a feed-forward network consisting of two dense layers with a rectified linear unit as activation function to carry out RiPP classification	32
Class specific	Pep2Path	MS-guided NRP/RiPP BGC identification	Bayesian algorithm NRP2Path matching short amino acid sequence tags to BGC-encoded NRPS assembly lines and employing colinearity index computation to interpret multiple BGC assignments; RiPP2Path matching putative RiPP amino acid sequence tags to the translation frames of a set of (meta)genomic sequences for precursor peptide identification	33
Class specific	ARTS (Antibiotic Resistant Target Seeker) 2.0	Antibiotic-resistant gene identification	antiSMASH-based detection of BGCs for resistance gene identification in (meta)genomic sequences; TIGRFAM protein model for core gene detection; resistance gene and core gene screening based on physical proximity, and detection of gene duplication and horizontal gene transfer events	34
Class specific	BAGEL4 (BActeriocin GEnome mining tooL)	Bacteriocin identification	Protein motif search BLAST against core peptides in the bacteriocin database; Glimmer-based ORF call; BLAST against annotation database; TransTermHP terminator prediction; motif-based promoter prediction	35
Class specific	RODEO (Rapid ORF^*b*^ Description and Evaluation Online)	Lasso peptide BGC identification	Profile HMM local genomic analysis; precursor peptide/structure prediction by a combination of heuristic scoring, motif analysis, and SVM^*b*^ classifier	36
Class specific	RiPPquest	MS-guided lanthipeptide (RiPP category) BGC identification	Pfam domain-based lanthipeptide gene clusters identification; prediction of MS/MS lanthipeptide spectra corresponding to core peptides predicted according to biosynthetic transformations and gas phase reactions in lanthipeptides; matching between predicted MS/MS lanthipeptide spectra and MS/MS spectra obtained by microbial extracts analysis; peptide homologs identification by spectra alignment	37
Class specific	DeepRiPP	MS-guided RiPP BGC identification	Bipartite algorithms adapted from natural language processing for identification of precursor peptides and their cleavage prediction (NLPPrecursor); Basic Alignment of Ribosomal Encoded Products Locally (BARLEY) combining retrobiosynthetic processing of known RiPP structures with local alignment to genomic sequences for candidate RiPP novelty computation; Computational Library for Analysis of Mass Spectra (CLAMS) integrating MS information to identify putative RiPPs within metabolomics datasets	38
Class specific	evoMining 2.0	NP biosynthetic enzyme identification	BLASTP-based search for expansion and recruitment events in enzyme families; phylogenies inference by approximately maximum-likelihood trees for assignment of metabolic origin and fate to enzyme family members	39
Class specific	decRiPPter (Data-driven Exploratory Class-independent RiPP TrackER)	RiPP BGC identification	Support vector machine classifier for RiPP precursor identification; pan-genomic analyses for assessment of their location within operon-like structures encoding accessory genes in a genus; evolutionary conservation- and enzymatic novelty-based ranking of precursors	40
Class specific	RiPPER (RiPP Precursor Peptide Enhanced Recognition)	RiPP BGC identification	RODEO-based identification of putative RiPP tailoring enzymes (RTEs); prodigal-short-based evaluation of the peptide-coding potential of regions surrounding RTEs; peptide optional similarity network approach for RiPP families identification	41
Class specific	NeuRiPP (Neural network identification of RiPP precursor peptides)	RiPP BGC identification	Deep neural network classifier trained on high-confidence ribosomally encoded precursor peptide sequences	42
Class specific	RiPP-PRISM	RiPP BGC identification	Profile HMM- and motif-based identification of RiPP BGCs, prediction of precursor peptide cleavage events, virtual reconstruction of post-translationally modifying reactions for combinatorial structure prediction across RiPP families	43
Class specific	MetaMiner	MS-guided RiPP BGC identification	Profile HMM-based identification of RiPP BGCs and their precursor peptides; target (via HMMer) and decoy putative RiPP structure databases construction; dereplicator-based matching between MS/MS spectra and the constructed target/decoy RiPP structures; spectral alignment for streamlining the peptide modifications identification	44
Class specific	RiPPMiner	RiPP BGC identification; prediction of NP structure	Support vector machine classifiers for prediction of RiPP class and cleavage sites; support vector machine or random forest classifiers for crosslinks prediction	45
Class specific	RiPPMiner-Genome	RiPP BGC identification; prediction of NP structure	Profile HMM-based RiPP BGC identification; random forest classifiers and support vector machine classifier for the prediction of precursor peptides, leader cleavage sites and crosslinks	46
Class specific	SANDPUMA (Specificity of Adenylation Domain Prediction Using Multiple Algorithms)	Non-ribosomal peptides (NRPS) adenylation domain specificity	Ensemble method based on active site motif sequence signatures, support vector machines, profile hidden Markov models, phylogenetically driven algorithm (prediCAT) calculating a confidence score for each A-domain based on comparative metrics against A-domains of known specificity	47
Class specific	NRPSPredictor2	NRPS adenylation domain specificity	Transductive support vector machines based on sequence and structural information about the active site of the adenylation domain	48
Class specific	CLUSEAN (CLUster SEquence ANalyzer)	Polyketide (PK)/NRP BGC annotation	Analytical workflow built on basic annotation (BLAST), protein domain identification against generalist profile databases (Pfam) and specialized databases for the identification of domains and conserved motifs of PKS/NRPS enzymes, classification of C-domain types and prediction of the specificity of NRPS adenylation domains	49
Class specific	BiosyntheticSPAdes	PK/NRP BGC identification	SPAdes- or metaSPAdes-based (meta)genomic assembling; identifying domain edges in the assembly graph; BGC subgraph extraction from the assembly graph; restoring collapsed domains in the assembly graph; constructing the scaffolding graph; constructing putative BGCs by solving the Rural Postman problem in the scaffolding graph	50
Class specific	NRPminer	MS-guided NRP BGC identification	antiSMASH for NRPS BGC prediction; NRPSpredictor2 for prediction of amino acid for each A-putative NRP structures domain; delineation of NRPS assembly lines accounting for modification enzymes; construction of putative NRP structures; matching between predicted NRPs and experimental spectra with score assignment	51
Class specific	Nerpa	Structure-guided NRP BGC identification	Retro-biosynthesis-based transformation of input structures into monomer graphs; generation of linear representations of monomer graphs; antiSMASH-based NRPS BGC prediction; BGC processing and heuristics-based generation of BGC monomer sequence according to collinear or non-collinear NRPS assembly lines; global alignment of NRP and BGC monomer sequences	52
Class specific	GRAPE (Generalized Retrobiosynthetic Assembly Prediction Engine)	Structure-guided PK/NRP assembly prediction	Retro-biosynthesis of PK and NRP by macrocycles opening, heterocycles opening, monomer linkages removal, identification of tailored additions, and PK processing	53
Class specific	SBSPKS (Structure Based Sequence analysis of PKS and NRPS)	Structure-guided PK/NRP BGC identification	Search for PKs and NRPs chemically similar to query molecule in SMILES format; search for tailoring reactions; linking chemical and genomic space	54
Class specific	GARLIC	Structure-guided PK/NRP BGC identification	Global alignment between monomers from antiSMASH-based BGC cluster predictions and GRAPE-based small molecule breakdowns (fatty acyl units, sugars, amino acids, and carboxylic acids) by random sampling of permutations	53
Miscellaneous	BiG-MAP (Biosynthetic Gene cluster Meta’omics Abundance Profiler)	BGC abundance and expression profiling	Mutual sequence similarity-based redundancy filtering on predicted BGCs (e.g., antiSMASH); BiG-SCAPE for BGCs clustering into GCFs; Bowtie2-based read mapping to non-redundant BGCs; differential abundance/expression analysis by parametric zero-inflated Gaussian distribution mixture model (ZIG model) or non-parametric Kruskal-Wallis test	55
Miscellaneous	BIG-SLiCE (Biosynthetic Genes Super-Linear Clustering Engine)	BGC clustering	BGC vectorization for the transformation of input BGCs into numerical feature vectors based on boolean values and bit scores of hits obtained querying BGC gene against Pfam and sub-Pfam profile hidden Markov models; superlinear clustering of BGCs into GCFs	56
Miscellaneous	BIG-SCAPE (BIosynthetic Gene Similarity Clustering and Prospecting Engine)	BGC clustering	Exploration of antiSMASH or MIMiB BGC sequence similarity networks based on combined metrics (Jaccard index, adjacency index, and domain sequence similarity index); metrics weights calibration for accounting for different evolutionary modes of BGC classes; affinity propagation clustering algorithm	57
Miscellaneous	CAGECAT (CompArative GEne Cluster Analysis Toolbox)	BGC comparative analysis	Search module relying on cblaster, which utilizes remote BLAST searches and accelerated local hidden Markov models; visualization module relying on the clinker pipeline	58
Miscellaneous	ModulesDetection	BGC module detection	Biosynthetic module identification in antiSMASH-predicted BGCs by orthoMCL-based evolutionary analysis and network analysis where nodes are clusters of orthologous genes, and edges are drawn based on adjacency and colocalization interactions	59
Miscellaneous	CORASON (CORe Analysis of Syntenic Orthologs to prioritize Natural product biosynthetic gene clustersBGC)	BGC phylogenetic analysis	Homology analysis of query genes in input BGCs (via antiSMASH and MIBiG); identification of the core genes in the genomic contexts harboring the identified homologous genes; multi-locus approximate-maximum-likelihood phylogenetic tree for prediction of clades synthesizing structurally different molecular products	57
Miscellaneous	NaPDoS2 (Natural Product Domain Seeker)	PK/NRP BGC phylogenetic analysis	Phylogeny-based classification scheme of ketosynthase and condensation domains	60
Miscellaneous	LASSOHTP	Lasso peptide structure prediction	Translation of input lasso peptide sequences and annotations (ring, loop, and tail) into conformational ensembles by scaffold construction, random mutagenesis, and molecular dynamics	61

^*a*^ For each software tool, the table reports the purpose(s) and a summary of its algorithmic components.

^*b*^ SVM, support vector machine; ORF, open reading frame; NP, natural product; MS, mass spectrometry.

**TABLE 2:** Overview of BGC-related databases^*a*^

BGC database	BGC type	General features	Reference
MlBiG (Minimum Information about a Biosynthetic Gene cluster) v.3.0	Experimentally validated BGC database	Accession mode: web interface. Query options: simple query by keyword; complex query builder. Construction method: manual curation. Additional notes: researchers are enabled to submit new BGC data to the database, adhering to MIBiG standards.	62
antiSMASH Database v.4.0	Computationally predicted BGC database	Accession mode: web interface, API. Query options: query by ribosomally synthesized and post-translationally modified peptide precursor based on NCBI BLAST+; search by protein sequence based on DIAMOND; search by NRPS/PKS module; search by single/multiple categories. Construction method: automated prediction of genomes obtained from NCBI RefSeq database by antiSLASH (v.7.1)	63
IMG-ABC (Integrated Microbial Genomes Atlas of Biosynthetic gene Clusters) v.5.0	Computationally predicted and experimentally validated BGC database	Accession mode: web interface. Query options: query by compound name, genome or accession ID, secondary metabolite ID, BGC ID or gene ID, comment, collection, taxonomy search, sequence similarity, and complex query builde. Construction method: automated prediction by antiSMASH v.5.0 and MiBIG content integration. Additional notes: researchers enabled to submit new BGC data to the database through specified system	64
ABC-HuMi (Atlas of Biosynthetic gene Clusters in the Human Microbiome)	Computationally predicted BGC database	Accession mode: web interface. Query options: query by user-provided nucleotide sequences based on BLAST+; query by translated nucleotide sequences based on tBLASTn; cblaster enabling the search of custom BGCs; filtering by metadata (taxa, body site, and product). Construction method: automated prediction of BGCs from MAGs from JGI GEM, isolates and MAGs from EMBL-EBI MGnify Catalogs and metagenomes from HMP using antiSMASH v.7.0	14
BIG-FAM	Computationally predicted GCF database	Accession mode: web interface. Query options: multi-criterion GCF search and GCF annotation of user-supplied BGCs. Construction method: automated prediction of GCFs by BiG-SLICE clustering of BGCs predicted by antiSAMH v5.1.1	65
sBGC-hm	Computationally predicted BGC database	Accession mode: web interface. Query options: query by cluster, family, organism, Genbank accession, and type. Construction method: automated prediction of BGCs from genomes obtained from the HumGut database and the HMP using antiSMASH v6.1.1	15
BGC Atlas	Computationally predicted BGC database	Accession mode: web interface. Query options: query by user-provided BGC sequence. Construction method: automated prediction of BGCs from genomes obtained from the MGnify database using antiSMASH v6.1.1 and subsequent clustering using BiG-SLiCE	66

^*a*^ For each BGC-related database, the table reports the experimental and/or computational origin of the included BGCs, accession modes, query options, and a summary of its construction method.

Current efforts to discover and catalog BGCs in the human microbiome mostly rely on traditional heuristics derived from the accumulated knowledge of specialized metabolic pathways, such as peptide modifications, acyltransferase activities, and adenylation domain substrate specificities. These approaches are often supported by supervised machine learning models, which are trained on heuristic-based data sets to identify BGCs. However, the emergence of protein language models (67, 68) has led to a paradigm shift in this field. These models inherently learn the structural and functional constraints encoded in the billions of protein sequences they are exposed to. The information derived from these models can then be represented as mathematical embeddings, enabling their application to specific tasks of interest, such as the accurate prediction and functional annotation of BGCs.

**Fig 2:**
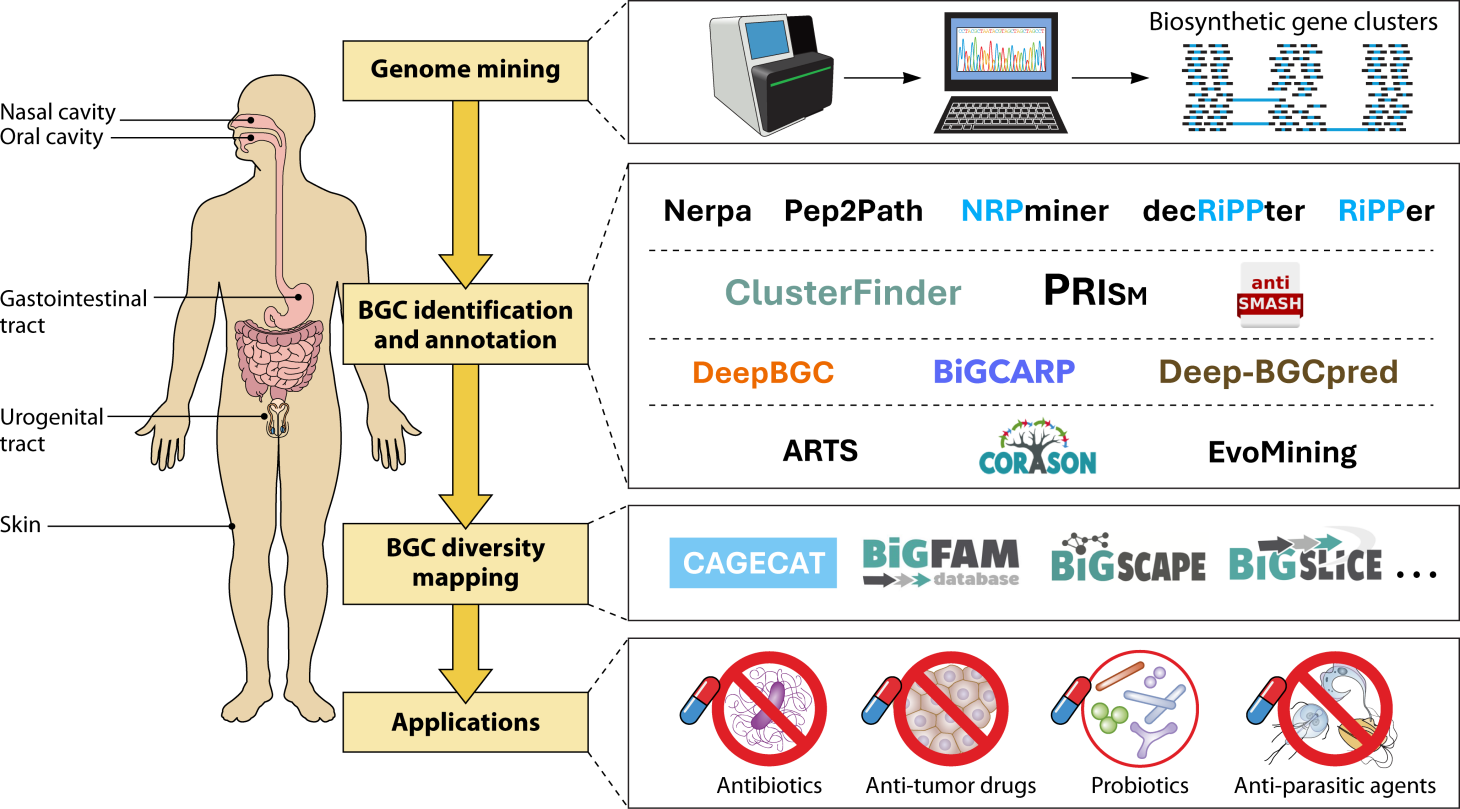
Decoding the biosynthetic diversity of the human microbiome through sequence-based modeling. Outline of sequence-based software tools for the characterization of BGCs endowed with applicative interest in (meta)genomes.

Against this backdrop, the present review provides a synopsis of both established and emerging methodologies for mining the human microbiome in the context of gene clusters responsible for synthesizing secondary metabolites (Fig. 2). We begin by detailing the primary BGC classes and their defining biosynthetic traits. To further elucidate these concepts, we then examine the BGC classes identified to date within human microbiome genomes and metagenomes, providing meaningful examples of biologically active compounds with relevant functional activities. Lastly, we analyze computational sequence-based tools designed for BGC detection, focusing on their algorithmic frameworks, the implications on inferred gene clusters, their suitability for analyzing single genomes and/or metagenomes, and their ability to characterize previously unclassified BGC classes.

Overall, this review provides researchers with a practical guide to the fast-evolving field of microbiome-derived natural product discovery, underscoring the need for better computational tools capable of integrating multi-omic data.

## BGC CLASSES

**Fig 3:**
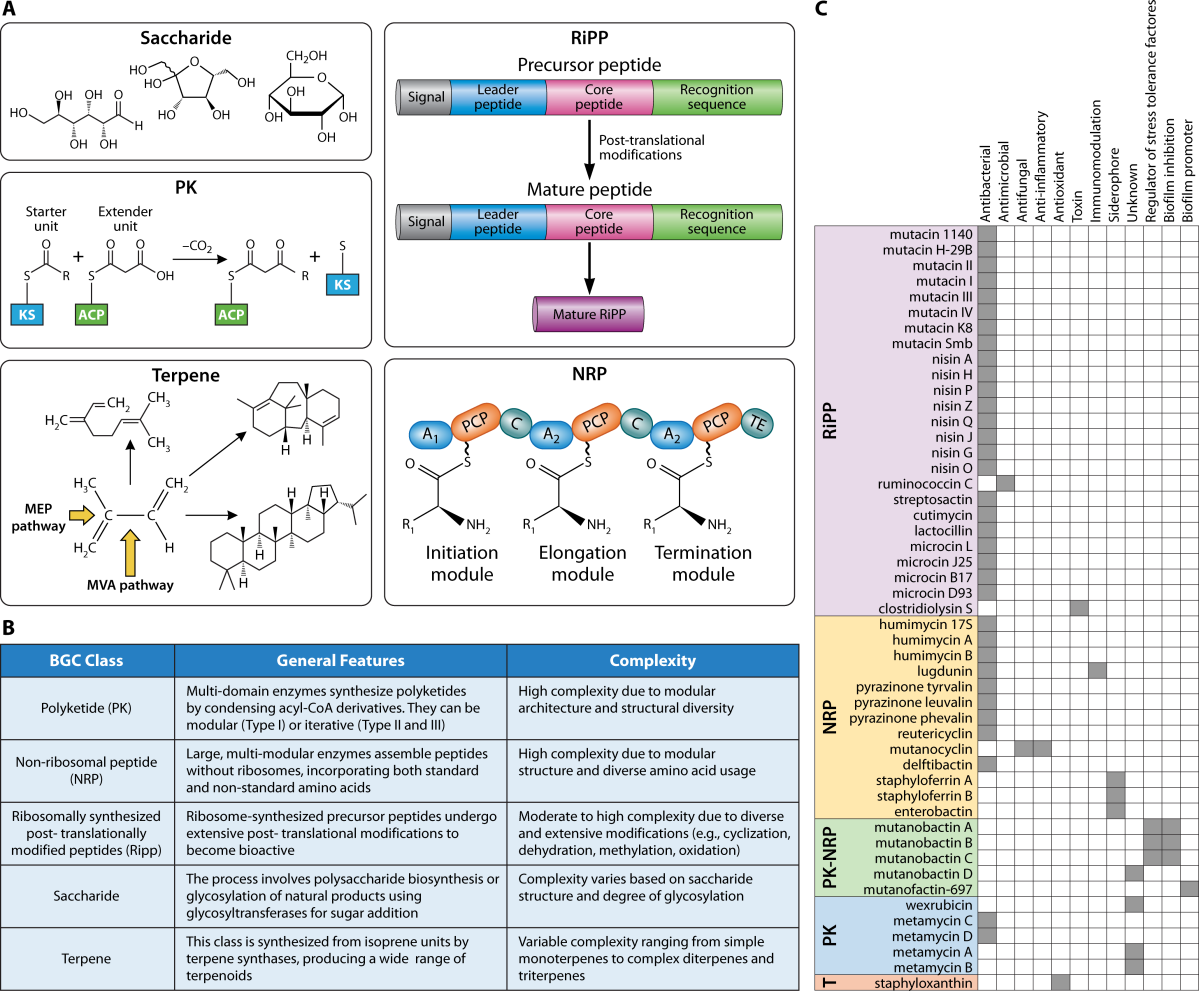
Biosynthesis of main secondary metabolite classes. (**A**) The panels present an overview of common features in the biosynthetic pathways of major BGC classes. Saccharide biosynthesis relies on monosaccharides such as glucose, fructose, and galactose, which serve as the fundamental building blocks that are enzymatically linked to form carbohydrates ranging from simple disaccharides to complex polysaccharides. The polyketide (PK) biosynthesis is carried out by modular PK synthases which consist of linearly assembled modules. Each module contains an acyl carrier protein (ACP) domain and a ketosynthase (KS) domain, along with a variable number of optional domains. During PK chain elongation, an ACP-bound acyl group condenses with a KS-bound substrate, releasing CO_2_ and forming an elongated chain on ACP. Ribosomally synthesized and post-translationally modified peptide (RiPP) biosynthesis starts with a precursor peptide consisting of a signal sequence, a leader peptide at the N-terminus of an unmodified core peptide, and a recognition sequence. The signal and the recognition sequence are optionally present in the precursor peptide. The precursor peptide undergoes post-translational modification, followed by leader peptide removal, resulting in the mature bioactive peptide. NRPS biosynthesis operates through modules containing adenylation (A), condensation (C), peptidyl carrier protein, and thioesterase domains, which sequentially activate, elongate, and release the peptide chain. Terpene biosynthesis relies on isoprene units which are produced through either the methylerythritol phosphate (MEP) or the mevalonate (MVA) pathway as universal precursors that are enzymatically assembled and modified to generate diverse terpenes. (**B**) The table summarizes the general features of the BGCs depicted in panel **A**, highlighting the biosynthetic mechanisms that contribute to the complexity of secondary metabolites biosynthesis. (**C**) The heatmap shows secondary metabolites genetically encoded by BGCs identified in human-associated microorganisms. The secondary metabolites are grouped according to their BGC class and functionally annotated by the broad categories depicted in the legend.

The Minimum Information about the Biosynthetic Gene Cluster (MIBiG) serves as a reference repository for manually curated data on secondary metabolites, currently exceeding 3,000 entries. Among these, roughly 50% are associated with over 3,600 documented biological activities, and more than 85% are linked to 5,000 distinct chemical structures (62). In this context, this review focuses on the predominant BGC classes found within the human microbiome, as cataloged in specialized databases (14, 15), and narrows the discussion to key biosynthetic traits (Fig. 3A and B) necessary to understand their functional relevance and the principles inspiring supervised BGC mining tools.

Non-ribosomal peptides (NRPs) constitute an important class of secondary metabolites synthesized by non-ribosomal peptide synthetases (NRPSs), which are large enzymatic complexes distinct from ribosomes (69). NRPSs function as modular assembly lines, where each module adds a specific amino acid to the growing peptide chain. Each NRPS module typically consists of at least three core domains: (i) the adenylation (A) domain, responsible for selecting and activating the amino acid to be lined up (70); (ii) the thiolation (T) domain, which allows the activated amino acid to travel within and between modules; and (iii) the condensation (C) domain, which catalyzes the peptide bond formation between the amino acid and the elongating chain (71). Because they are ribosome independent, NRPSs can introduce not only proteinogenic but also non-proteinogenic amino acids into the produced peptides. Post-assembly modifications by specialized tailoring enzymes further increase this diversity. The remarkable variability in NRPs is thought to arise from (i) the modular architecture of NRPSs, which allows autonomous operation of each module and combinatorial amino acid incorporation; (ii) the broader substrate range of monomers compared to ribosomal peptides; and (iii) the extensive peptide modifications during and after chain assembly.

Ribosomally synthesized and post-translationally modified peptides (RiPPs) are initially synthesized as precursor peptides, which are typically larger than the final products resulting from the maturation process. The modifying processes release the precursor peptides from the structural and functional constraints characteristic of ribosomal products while restricting conformational flexibility to enhance target recognition and increase stability (72). In general, a precursor peptide consists of an N-terminal leader peptide and a C-terminal core peptide (73). Even though the roles of the leader peptide can vary depending on the RiPP sub-class, its primary functions include facilitating the recognition of the precursor peptide by the RiPP post-translationally modifying enzymes via the RiPP precursor recognition element (RRE) and promoting the export of the RiPP out of the cell (74).

The core peptide undergoes post-translational modification by the RiPP biosynthetic machinery, is proteolytically cleaved from the leader peptide to yield the mature RiPP, and is subsequently exported out of the cell by transporters. RiPPs encompass around 40 sub-classes (75), including polycyclic peptide antibiotics, bacteriocins, cytolysins, amatoxins, cyclotides, microviridins, and conopeptides (74). These examples point to the significant chemical diversity among RiPPs, underscoring their potential for translational applications.

Polyketides (PKs) are versatile in structure and function (76). Examples of PK compounds that have entered the drug market include erythromycin, known for its antimicrobial properties, rapamycin, an immunosuppressant, and doxorubicin, used as an anticancer agent. Such diversification is ascribable to the intrinsic adaptability of PK biosynthesis, which functions through several critical stages. The assembly of the PK carbon scaffold, driven by the choice of the starter units and—to a limited degree—extender modules, provides the basic framework for diversity. In addition, catalytic domains within the biosynthetic machinery act as decision gates that determine the final structure and release of the product. Tailoring enzymes further enhance chemical complexity through scaffold modifications, such as cyclization and dimerization of the β-keto-acyl carbon chain, as well as other changes that affect PK activity.

Terpenes, a ubiquitous class of natural compounds, are synthesized from two building blocks: isopentenyl diphosphate and its isomer dimethylallyl diphosphate. These precursors are synthesized through either the methylerythritol phosphate (MEP) pathway or the mevalonate (MVA) route. Most bacteria predominantly utilize the MEP pathway, while some employ the MVA pathway or a combination of both, reflecting metabolic adaptations to diverse ecological niches (77, 78).

## BGC DISCOVERY IN THE HUMAN MICROBIOME

Breakthroughs in molecular techniques, such as amplicon sequencing, whole microbial genome sequencing, and metagenomics, have substantially expanded our knowledge of the complex microbial communities inhabiting various human body sites (79). The resulting sequencing data, assembled within collaborative frameworks, such as the US National Institutes of Health-funded HMP (80, 81) and the Human Gastrointestinal Bacteria Genome Collection (82), as well as broader initiatives like the Genomic Catalog of Earth’s Microbiomes (JGI GEM) (83) and the EMBL-EBI MGnify catalogs (84), have been extensively mined to investigate BGC content in human-associated genomes (12).

A healthy human microbiome features an extensive assortment of BGCs. For instance, the ABC-HuMi database currently catalogs 19,218 BGCs computationally predicted from the (meta)genomic sequences derived from 14 body sites, including the gut, oral, skin, respiratory, and urogenital systems. This biosynthetic wealth is further underscored by the classification of BGCs into 8,989 gene cluster families (GCFs) and 294 gene cluster clans. The main BGC classes identified are saccharides, RiPPs, NRPs, PKs, and terpenes, each variably distributed across body sites (12, 14). In this regard, the gut (8, 12, 85, 86) and oral (13, 87, 88) microbiomes have been the most extensively analyzed for their BGC content.

Saccharides constitute the most abundant BGCs in the human microbiome, with significant enrichment observed in gut and oral samples (89). One of the best-characterized examples is polysaccharide A (90), encoded by the ubiquitous gut microorganism *Bacteroides fragilis*, which is renowned for its immunomodulatory activity.

BGCs encoding specialized metabolites have consistently proven to be a reliable source of compounds with impactful biomedical applications. Examples include the PK antibiotics erythromycin and tetracycline; the NRP antibiotics penicillins and cephalosporins; and the glycopeptides from the vancomycin family (91). Despite this success, the sequence-guided discovery of specialized metabolites encoded in the human microbiome has progressed at a relatively slow pace, yielding a limited number of bioactive compounds derived from the identified BGCs.

Most specialized metabolites that have been characterized so far belong to the RiPPs type (Fig. 3C), encompassing lanthipeptides (92), sactipeptides (93, 94), thiopeptides (12), and microcins (95). RiPPs are particularly interesting antimicrobial compounds due to their narrow-spectrum activities and their ability to employ multiple simultaneous mechanisms of action, thereby reducing off-target effects and minimizing the risk of resistance development (96). However, none of the RiPPs identified from human microbiome-derived metagenomic sequencing data have yet been approved for clinical use in humans. A promising candidate is LFF571, a derivative of thiopeptide GE2270-A, produced by the vaginal isolate *Lactobacillus gasseri* JV-V03, which showed potent activity against *Clostridium difficile* infections and successfully completed a phase II clinical trial (97). Another notable compound is lactocillin, identified in the vaginal isolate of *L. gasseri* JV-V03 as well during a systematic analysis of BGCs in the HMP metagenomic samples. Lactocillin has been proposed to play a role in protecting the vaginal microbiota against pathogen invasion (12).

Mining metagenomic sequences has also uncovered a large family of 47 NRP BGCs, widely distributed in HMP stool samples and highly prevalent in the gut microbiome (12). Preliminary analysis of these gene clusters in RNA-seq data sets from stool samples has revealed robust expression levels for at least one BGC in nearly all samples. A subset of the identified BGCs was shown to be peptide aldehydes, which bear protease inhibitory activity with pronounced selectivity toward a subset of cathepsins involved in immune responses (98). Conversely, relatively few polyketides have been characterized from human microbiome sequences. One of them is the potent genotoxic colibactin (99), which is encoded by various *Escherichia coli* strains belonging to the B2-phylogroup and has been strongly implicated in the development of colorectal cancer (100).

Albeit scarcely applied to human microbiome-related BGCs, approaches pairing sequence-based computational imputation of BGCs with synthetic biology have led to the discovery of molecules with promising bioactivity (101, 102). For example, the discovery of two closely related NRPS BGCs by mining the human microbiome sequences in the HMP (80) and HOMD (103) repositories led to the chemical synthesis of two humimycins, which were found to be broadly active against *Firmicutes* and, to a lesser extent, *Actinobacteria*, the dominant phylum in the human gut microbiome (104). More recently, lasso peptides, discovered in human commensal organisms, have been refactored using *E. coli* codon-optimized genes for *E. coli* expression, resulting in the production of novel lasso peptides, whose bioactivities warrant further investigation (11).

Despite these notable examples, the discovery of the chemical repertoire encoded by the human microbiome remains significantly underdeveloped compared to its well-documented taxonomic richness, as corroborated by multiple human microbiome studies (13, 90). This disparity calls for the systematic interrogation of the microbiome as a source of pharmacologically and, more broadly, biotechnologically valuable molecules. A wide variety of genome mining approaches, which we address in detail below, have become readily accessible (105). Moreover, with the growing availability of matched genomic and metabolomic data sets on platforms such as the Paired Omics Data Platform (106), the prioritization, functional analysis, and structural characterization of BGCs synthesizing natural products can now be considerably enhanced by linking mass spectral data to gene clusters (30, 107, 108).

## BGC IDENTIFICATION AND ANNOTATION

BGC discovery involves a wide range of information processing tools designed to automate the identification and functional annotation of BGCs from genomic data. This crucial task presents significant challenges as it entails solving several complex sub-tasks that require innovative algorithmic solutions. Specifically, BGC discovery encompasses not only the identification of BGCs in (meta)genomic sequences but also their classification, dereplication, prioritization, connection with metabolite data, and functional characterization.

This review focuses on the sequence-based identification and classification of gene clusters in different settings (Tables 1 and 3).

**TABLE 3:** Mapping frequently asked questions concerning BGC identification to representative software tools^*a*^^,^

Tool	URL	Are you interested in any BGC class?	Are you searching for specific BGC classes?	Are you searching for a tool assisted by biosynthetic logic?	Are you searching for a supervised ML^*b*^ tool?	Are you searching for an NLP-inspired ML tool?	Are metagenomic data available?	Would you like to link BGCs to NPs?	Would you like to compare BGCs?	Would you like to avail yourself of evolutionary principles?	Are you interested in analytics workflows?	Are structural data available?	Are expression data available?
Antibiotics and Secondary Metabolite Analysis SHell (antiSMASH)	https://antismash.secondarymetabolites.org	x		x	x		x						
Antibiotic- Resistant Target Seeker	http://arts.ziemertlab.com		x	x	x		x			x			
BAGEL4	http://bagel4.molgenrug.nl		x		x		x						
BGCFlow	https://github.com/NBChub/bgcflow	x		x	x		x		x	x	x		
BGC-Prophet	https://github.com/HUST-NingKang-Lab/BGC-Prophet	x			x	x							
BiGCARP	https://github.com/microsoft/bigcarp	x			x	x							
BiG-MAP	https://github.com/medema-group/BiG-MAP	x		x	x		x				x		x
BiG-SCAPE	https://bigscape-corason.secondarymetabolites.org/	x		x	x		x		x				
BiG-SLiCE	https://github.com/medema-group/bigslice	x		x	x		x		x				
BiosyntheticSPADES	https://genome.cshlp.org/content/suppl/2019/07/24/gr.243477.118.DC1		x				x						
CLUSEAN	https://bitbucket.org/tilmweber/clusean/src/master/		x	x									
ClusterFinder	https://github.com/petercim/ClusterFinder	x			x		x						
decRiPPter	https://github.com/Alexamk/decRiPPter		x		x					x			
DeepBGC	https://github.com/Merck/deepbgc	x			x	x	x						
Deep-BGCpred	https://github.com/pmobio/Deep-BGCpred	x			x	x							
DeepRiPP	https://github.com/magarveylab/NLPPrecursor/tree/master		x		x	x			x				
EvoMining	https://github.com/nselem/evomining	x								x			
GARLIC	https://github.com/magarveylab/garlic-release	x		x	x		x	x				x	
GECCO	https://gecco.embl.de	x			x		x						
GRAPE	https://github.com/magarveylab/grape-release		x	x				x				x	
IsaBGC	https://github.com/Kalan-Lab/lsaBGC	x		x	x	x	x		x		x		
MetaMiner	https://github.com/ablab/npdtools		x	x	x		x	x					
Nerpa	https://github.com/ablab/nerpa	x		x	x		x						
NeuRiPP	https://github.com/emzodls/neuripp		x		x		x						
NPLinker	https://zenodo.org/records/4680579	x		x	x		x	x	x				
NPOmix	https://github.com/tiagolbiotech/NPOmix_python	x		x	x		x	x	x				
NRPminer	https://github.com/mohimanilab/NRPminer		x	x	x			x					
NRPSPredictor2	https://github.com/roettig/NRPSpredictor2		x		x		x						
Pep2Path	https://pep2path.sourceforge.net	x		x	x		x	x					
PRISM3	https://doi.org/10.1101/2023.05.23.540769	x		x	x								
PRISM4	http://prism.adapsyn.com	x		x	x		x						
RiPPER	https://github.com/streptomyces/ripper		x	x	x		x		x				
RiPPMiner	http://www.nii.ac.in/~priyesh/lantipepDB/new_predictions/index.php		x		x								
RiPPMiner-Genome	http://www.nii.ac.in/~priyesh/lantipepDB/new_predictions1/cyclizationPrediction.php		x		x								
RiPP-PRISM	https://doi.org/10.1101/2023.05.23.540769		x	x									
RiPPquest	http://gnps.ucsd.edu/ProteoSAFe/static/gnps-theoretical.jsp		x	x				x					
RODEO	https://webtool.ripp.rodeo/		x	x	x		x						
SANDPUMA	https://bitbucket.org/chevrm/sandpuma		x	x	x					x			
SBSPKS	http://www.nii.ac.in/sbspks2.html		x	x				x				x	
TaxiBGC	https://github.com/danielchang2002/TaxiBGC_2022		x				x	x					
TrRiPP	https://github.com/zzhongzz/TrRiPP		x		x	x	x						

^*a*^ The table displays common questions which arise when carrying out BGC genome mining along with the software tools serving as examples of the available solutions to answer the intended questions.

^*b*^ ML, machine learning.

### BGC-class independent genome mining

To identify putative BGCs from isolate genomes or metagenomes, two basic approaches can be employed: principled methods, which leverage accumulated knowledge on biosynthetic logic, and data-driven methods, which use machine learning and statistical models to infer patterns from large data sets. The former excels at predicting BGCs encoding known classes of biosynthetic enzymes, yielding low false positive rates, but its ability to discover novel classes is limited. Conversely, data-driven approaches facilitate the identification of previously unknown BGCs but often exhibit higher false positive rates for uncharacterized clusters and increased false negative rates for known ones.

Biosynthetic logic-assisted tools rely on the observation that BGCs often share common properties, particularly enzyme families responsible for catalyzing key biochemical transformations involved in specialized metabolite biosynthesis. Popular tools such as antibiotics and Secondary Metabolite Analysis SHell (antiSMASH) (21) and PRediction Informatics for Secondary Metabolomes (PRISM) (27) employ profile hidden Markov models (pHMMs) of protein domains generated from multiple sequence alignments to identify gene combinations encoding biosynthetic pathway signatures. The use of pHMMs is very reliable for identifying BGCs encoding many well-established types of biosynthetic machinery, including PKSs, NRPSs, and known classes of RiPPs. For instance, antiSMASH 7.0 (21) can recognize up to 81 BGC types. While both antiSMASH and PRISM generally provide very similar results, antiSMASH has increasingly emphasized functional and comparative genomic analyses, whereas PRISM has refined a combinatorial approach for chemical structure prediction, enabling automated matching with mass-spectral data.

Data-driven tools play a key role in discovering new BGC types, thereby expanding the range of natural products. Various procedures have been implemented to achieve this goal (Table 1). One pioneering method, ClusterFinder (20), avoids reliance on predefined biosynthetic gene profiles by relying on domain-based functional profiles. More specifically, ClusterFinder identifies gene clusters of both known and unknown classes using a two-state HMM-based probabilistic algorithm, where one hidden state corresponds to BGCs (BGC state) and the other represents non-BGC genome (non-BGC state). This approach converts a nucleotide sequence of a certain genome into a structured series of Pfam domains, assigning probabilistic scores to each domain based on its frequency distribution in BGC and non-BGC training data sets, while also considering the identities of adjacent domains for improved accuracy. After computing BGC probabilities for all domains processed by the algorithm, ClusterFinder identifies gene clusters as sets of genes separated by no more than one intervening gene and containing at least one domain with a BGC probability above a predefined threshold. Since the ClusterFinder procedure relies on functional domains, which can assemble in various ways to produce distinct natural products, it comes with minimal training set bias and is particularly effective in guiding the discovery of hybrid and novel BGC classes.

Evolutionary principles have been instrumental in guiding the development of several BGC detection tools. Some of them incorporate synteny analysis, taking advantage of the fact that biosynthetic genes are often physically clustered. Indeed, this analysis enables the detection of co-localized genetic loci that appear in phylogenetically related organisms but are absent from core genomes, making it an effective method for identifying BGCs (109).

Other evolution-based approaches do not rely on synteny analysis. A key example is EvoMining (39), which identifies metabolism-associated non-syntenic gene blocks based on the premise that genes involved in secondary metabolism originate as paralogs of primary metabolic enzymes. Indeed, an enzyme family can undergo accelerated evolution through duplication or horizontal gene transfer events, with retained gene copies conferring an evolutionary advantage by enabling secondary metabolite biosynthesis.

EvoMining requires a genome database, an enzyme database, and a database of natural product biosynthetic enzymes. Initially, EvoMining detects expansion and recruitment events within enzyme families. An expanded family consists of all enzyme copies retrieved by querying the genome database with the entries of the enzyme database. Subsequently, the expanded enzymatic family is cross-referenced with the database of natural product biosynthetic enzymes to identify its potential role in specialized metabolism. Enzyme families associated with expansion and recruitment events are then subjected to phylogenetic analysis. The ensuing phylogenetic trees are examined to pinpoint enzymes more closely related to those predicted to be involved in natural product biosynthesis rather than those engaged in central metabolism. Such enzymes are deemed putative candidates for novel functions in specialized metabolic pathways.

As briefly outlined in the previous section, HMMs are widely employed in various tools, including antiSMASH, PRISM, and ClusterFinder (Table 1). A known disadvantage of HMMs is represented by their inability to recognize position dependencies between genomically distant entities (110). DeepBGC was designed to address this algorithmic limitation by implementing a deep learning approach based on bidirectional long short-term memory (Bi-LSTM) recurrent neural networks and vector representations of Pfam protein family domains (pfam2vec), which inherently capture dependencies between both adjacent and distant entities in the genome (26). Furthermore, a prominent feature of DeepBGC is its post-processing stage, where a random forest classifier, trained and tested on the MIBiG database, enables the classification of putative BGCs based on their corresponding product classes and broad molecular mechanisms of action. Of note, the DeepBGC random forest classifier can detect the most influential Pfam domains, thereby providing a data-driven alternative to expert knowledge-based classification approaches that rely on human-defined rules.

Deep-BGCpred, inspired by DeepBGC, boasts several technical innovations to mitigate false positives and enhance model stability (23). This method consists of two stages: a Pfam protein family domain encoder and a stacked Bi-LSTM model. Unlike DeepBGC, Deep-BGCpred encodes not only Pfam domain identifiers into pfam2vec vectors but also integrates additional neural network vectors incorporating Pfam domain annotations, such as the domain summaries and high-level classification information. Similarly to DeepBGC, these embedding vectors are concatenated and fed into a stacked Bi-LSTM neural network, which predicts Pfam domain scores. These scores are then averaged per gene, and genes with scores exceeding a predefined threshold are grouped into BGCs. Finally, Deep-BGCpred employs a random forest classifier that assigns BCGs to one of eight categories consisting of a non-BGC class and seven BGC ones. To reduce the number of false positives in predicted BGCs, Deep-BGCpred enables the creation of negative samples using an augmentation technology that exploits the Pfam domain similarity network in the European Molecular Biology Laboratory (EMBL) database.

A source of model instability is the imbalance between the number of protein family domains in artificially created training data and the real genomic data. To address this inconsistency, Deep-BGCpred applies a sliding window procedure to the training data, allowing multiple Pfam sequence fragments to be derived from continuous Pfam sequences for improved Pfam score computation.

The language processing neural network BGC-Prophet has recently been introduced to detect both known and novel BGCs and classify their natural products (111). This tool is trained on thousands of genomes to learn gene location dependencies using the evolutionary scale modeling (ESM) method, which relies on the fact that protein sequences at the evolutionary scale offer a representation of biological structure and function. A subsequent fine-tuning phase ensures that the representational capacity of the embedding is specifically honed to grasp context information relevant to biosynthetic gene clusters. The embedding vectors are then input to a transformer encoder language model that employs a multi-head self-attention mechanism to speed up the training phase and improve accuracy in BGC prediction and multi-label classification. The increase in computational speed makes BGC-Prophet suitable for processing also metagenomic data sets and pan-phylogenetic screenings.

The machine learning tools discussed so far are supervised models. As such, their predictive quality is tied to the accuracy and depth of the training data sets. The difficulty of accessing massive amounts of high-quality and balanced labeled data is known to significantly impact model performance. This limitation can be addressed by self-supervised protein language models, which are gaining growing attention. In such approaches, models are pretrained on large volumes of unlabeled data to capture fundamental knowledge underlying proteins in such a way that they can then use the captured principles to carry out diverse analytical tasks. Alternatively, they can be fine-tuned on downstream supervised tasks for enhanced specificity. Although still in the early stages of application, self-supervised masked language models have recently been employed for BGC identification. These models train neural networks to reconstruct missing tokens in a corrupted sequence or predict the next element in a sequence based on preceding ones. One such method is Biosynthetic Gene Convolutional Autoencoding Representations of Proteins (BiGCARP) (24), which models BGCs as chains of functional protein domains and uses the ESM-1b transformer to obtain pretrained embeddings of these protein domains. BiGCARP trains a convolutional neural network (CNN)-based masked language model, built upon ByteNet (112) and CARP (113), on such domains to acquire BiGCARP, which can be used to predict BGCs and classify their products. As mounting evidence suggests that the adoption of self-supervised training schemes can effectively expand the detection capabilities of BGCs beyond known classes, further investigation into self-supervised language modeling approaches is warranted. The computational advances in BGC prediction enabled by CNNs, which—unlike transformers—scale linearly with the input sequence length, highlight the potential for further exploration of alternative CNN architectures.

#### Mapping BGC diversity

The substantial diversity and distribution of BGCs and their associated secondary metabolites are now being recognized thanks to the utilization of genome mining techniques across various levels of biological organization. Sequence-similarity-based networks have eased the exploration of relationships among BGC architectures, leading to the identification of biosynthetic GCFs. Organizing BGCs into GCFs can provide valuable insights into the recognition and classification of newly identified BGCs. Furthermore, it can help link the identified BGCs to their corresponding peptide products by detecting statistically significant correlations between the presence of GCF members in genomic data sets and that of chemotypes in MS data sets (114).

Conventional sequence similarity-based approaches start by comparing BGC protein sequences, an all-vs-all problem that scales quadratically with increasing data set size. Given the sheer volume of BGCs, such methods require lengthy CPU time, making them impractical for routine research. In addition, sequence-similarity-based methods may incorrectly quantify the similarity between fragmented gene clusters, which are common in metagenomic and large-scale pan-genome sequencing data sets generated through short-read technologies.

The Biosynthetic Gene Similarity Clustering and Prospecting Engine (BIG-SCAPE) tool is a sequence-similarity-based method employed to reconstruct GCFs. It addresses the aforementioned shortcomings by reframing the all-vs-all sequence comparisons into Pfam-based pairwise comparisons of pHMMs to obtain a similarity network. GCFs are then extracted using an affinity propagation clustering algorithm (57). Moreover, BIG-SCAPE becomes more informative by facilitating the analysis of evolutionary relationships among BGCs through CORe Analysis of Syntenic Orthologs to prioritize Natural Product Biosynthetic Gene Clusters (CORASON), which computes high-resolution multi-locus phylogenies of BGCs within and across GCFs (57).

Unlike BIG-SCAPE, which relies on pairwise comparisons to build GCF networks, Biosynthetic Genes Super-Linear Clustering Engine (BiG-SLiCE) queries a library of curated pHMMs with input BGCs. The presence or absence of hits, along with their bit scores, is used to generate numerical feature vectors corresponding to the input BGCs. BiG-SLiCE then projects these vectors into Euclidean space where it runs a partitional clustering algorithm in a near-linear time complexity. This computational efficiency allows BiG-SLiCE to handle increasingly large data sets as they become available (56).

### Metagenomic BGC mining

The emergence of shotgun metagenomic sequencing has revolutionized BGC prediction, extending its application beyond culture-dependent genomes to the broader biosynthetic potential of interacting microbial communities. Whether heuristic or machine learning based, most of the existing tools assume that each BGC is confined within a single contig in genome or metagenome assemblies. While this assumption presents challenges for sequenced microbial genomes—where BGCs are often scattered through multiple contigs—it is even less feasible in shotgun metagenomics, where contigs are often fragmented and short.

An alternative approach is represented by exploiting genome assembly graphs, exemplified by biosyntheticSPAdes (50), a tool that allows users to assemble NRPS BGCs, PKS BGCs, and mixed NRPS-PKS BCCs from assembly graphs generated by SPAdes (115) and metaSPAdes (116) assemblers. The core premise of BiosyntheticSPAdes is that reconstructing the arrangement of biosynthetic domains within a BGC is often sufficient to predict the core scaffold of the secondary metabolite encoded by such a cluster. The algorithm identifies domain motifs along assembly graph edges, extracts BGC assembly subgraphs by selecting all edges within a preset distance from previously detected domain edges, and subsequently generates a scaffolding graph linking contigs with closely positioned domains. The final step consists of solving the Rural Postman problem—consisting of finding a closed walk traversing a given subset of edges with minimum total cost—to reconstruct putative BGC arrangements in the scaffolding graph.

The Metagenomic identifier of Biosynthetic Gene Clusters (MetaBGC) offers an assembly-independent framework for BGC detection in microbiomes by directly analyzing metagenomic reads, which are scored according to their alignment with predefined pHMMs (101). To reconcile the discrepancy between the full-length proteins used in pHMM training and the short metagenomic reads (typically ~100 bp long), MetaBGC transforms these models into segmented pHMMs (spHMMs). Metagenomic reads are then scored against the spHMMs and retained for further processing provided that their scores exceed predefined cutoffs. Since reads originating from the same BGC are reasonably expected to display similar coverage across metagenomic samples, biosynthetic reads are subsequently dereplicated, quantified, and binned according to their abundance profiles, ultimately facilitating BGC prediction. Despite the ability of MetaBGC to bypass biases caused by reliance on cultured isolates or metagenomic assemblies, its implementation is constrained by the need for fine-tuning multiple parameters for spHMM-based biosynthetic read identification, quantification, and clustering. Addressing this limitation through automated optimization and adaptive parameter selection may ameliorate the accuracy and scalability of BGC prediction in metagenomic data sets.

Another genome assembly-independent method is the Taxonomy-guided Identification of Biosynthetic Gene Clusters (TaxiBGC), which operates a structured three-stage workflow (25). First, it performs species-level taxonomic profiling of metagenomic samples. Second, it queries the TaxiBGC reference database to infer the presence of experimentally characterized BGCs in the identified species. Third, it evaluates the predicted BGCs by aligning the metagenomic reads to these clusters, ensuring they meet minimum criteria for the presence of a BGC gene and overall BGC coverage. Once validated, the corresponding secondary metabolites are retrieved from the TaxiBGC reference database. The MIBiG database provides annotated information on experimentally characterized BGCs and their associated products, which are stored in the TaxiBGC reference database.

As dictated by its pipeline design, the performance of TaxiBGC is strictly dependent on the accurate assignment of predicted BGCs to microbial species identified through metagenome taxonomic profiling, as well as the depth of structural and functional characterization available for known BGC classes. A key feature of the TaxiBGC method is its intrinsic ability to identify specific microbial species harboring the predicted BGC genes.

The Secondary Metabolite Gene Cluster Annotations using Neural Networks Trained on InterPro Signatures (SanntiS) tool represents a more recent machine learning-based approach suitable to both genomic and metagenomic data sets (117). It consists of an artificial neural network with a one-dimensional convolutional layer and a Bi-LSTM to classify BGCs based on representations learned from the MIBiG database.

Despite the advancements achieved through the computational approaches mentioned, a fundamental challenge remains in identifying yet-to-be-characterized BGC classes in metagenomic samples, as each tool ultimately relies on known BGC models. Consequently, the integration of unsupervised learning algorithms into BGC detectors is expected to boost our ability to fully understand the biosynthetic potential of microbial communities.

### BGC class-specific mining approaches

Besides general-purpose tools for BGC mining, a broad range of specialized computational approaches have been developed to help discover specific BGC classes. These tools leverage extensive knowledge of biosynthetic pathways, chemical scaffolds, and bioactivities to improve prediction accuracy. A comprehensive survey of the relevant literature, focusing on studies associated with tools that have been carefully curated and maintained over time, is summarized in Table 1. Several of these specialized tools serve as BGC class-oriented detectors, providing targeted solutions for RiPP-, NRP-, and PK-producing BGCs.

RiPPs are characterized by the absence of conserved biosynthetic features and extensive structural diversity, which makes them highly promising for drug discovery applications (118–120) but also particularly challenging to discover (121). As a result, genome mining for RiPP gene clusters has become a focal point in biomedicine and cheminformatics, leading to the development of several specialized tools.

One of the earliest tools is RiPP-PRISM (43), which extends the PRISM framework by incorporating: (i) motif assembly for precursor cleavage prediction, (ii) HMMs for detecting a broad range of RiPP classes, and (iii) virtual tailoring reactions to infer the chemical structure of RiPP products. In general, class-dependent RiPP genome mining relies on sequence similarity to known biosynthetic enzymes (75), whereas class-independent approaches leverage conserved RiPP RREs. For example, the RRE-Finder (122) combines pHMMs with a truncated HHpred pipeline (123) to facilitate the detection of divergent RRE sequences. Rapid ORF Description and Evaluation Online (RODEO) (36) can then analyze RRE-containing proteins, reducing false positives by identifying co-occurring ORFs encoding biosynthetic enzymes.

Another approach employed to identify first-in-class RiPPs is represented by RiPPER (41), which scans genomic regions flanking putative tailoring enzymes for ORFs with lengths consistent with known RiPP precursor peptides. RiPPMiner uses a support vector machine (SVM) classifier to distinguish RiPP precursor peptides from other small proteins, while also predicting RiPP classes, leader peptide cleavage sites, and potential crosslinks in the core peptide (45). RiPPMiner-Genome builds on this approach by accepting genomic sequences as input—instead of relying on RiPP precursor peptide sequences—and predicting crosslinked RiPP structures (46). This update was made possible by systematically revising BGC information for RiPPs with known chemical structures and updating the prediction rules for RiPP precursor identification, cleavage motifs, and core modifications.

Deep learning-based methods have also contributed to RiPP genome mining. For instance, DeepRiPP (38) adapts architectures from natural language processing, whereas NeuRiPP uses a neural network structure (42). Furthermore, TrRiPP (32) combines transformer encoders with Bi-LSTM layers to discriminate RiPP precursors from non-RiPP short peptides and classify RiPPs into subclasses. Since these tools rely on the precursor peptides for predicting RiPPs, they do not suffer from limitations in RiPP identification associated with fragmented assemblies or the presence of distantly encoded modification enzymes. As such, they are well suited for RiPP identification in metagenomic sequences.

The discovery of NRPs and PKs poses significant challenges due to the modular assembly of these systems (76, 124), the broad substrate specificity of adenylation domains responsible for amino acid recognition and activation, and their extensive post-assembly modifications. Addressing this biosynthetic complexity has led to the emergence of tools that often couple general-purpose BGC mining tools, such as antiSMASH, with NRPS- and PKS-specific predictors. Examples include NRPSpredictor2 (48), the NRPyS library (125), and SANDPUMA (47), all of which are capable of predicting the substrate specificity of the adenylation domains. In addition, NRPminer (51), Nerpa (52), and SBSPKS (54) employ distinct computational strategies to integrate (meta)genomic and metabolomic data, with the common goal of linking predicted BGCs to their corresponding NRPs and PKs.

Unlike the previous tools, NaPDoS (60) utilizes a phylogeny-based classification of ketosynthase (KS) and condensation domains to infer PK and NRP biosynthetic potential. Since it does not require fully assembled BGCs, it becomes especially useful when analyzing large metagenomic and PCR amplicon data sets.

Finally, DeepT2 (126) applies principles from protein natural language processing to predict bacterial type II PKs. It converts protein sequences of key ketosynthases into vector embeddings using EMS-2 and employs semi-supervised learning to associate KS embeddings with the PK class labels, facilitating the prediction of both known and novel type II PKs.

## CONCLUSIONS

The present review provides a detailed assessment of sequence-based BGC mining tools that have significantly advanced our understanding of the biosynthetic potential of both isolate genomes and complex microbial communities across diverse ecological environments (127). Based on existing studies, the systematic characterization of the specialized metabolites produced by human-associated microorganisms has not only revealed the mechanistic interplay between human microbiome and health—an association that continues to gain recognition (128–130)—but has also contributed to the discovery of bioactive compounds with promising biomedical applications (6).

The genetic basis of specialized metabolites in the human microbiome has been extensively investigated using established tools like ClusterFinder and antiSMASH. Nonetheless, the rapid accumulation of (meta)genomic sequencing data has far outpaced our ability to gain new insights into gene clusters synthesizing biologically active molecules. To address this gap, it is worth pursuing the integration of complementary computational methodologies—such as the unsupervised machine learning algorithms outlined in this review—to enhance discovery rates and facilitate the identification of novel biosynthetic pathways.

As our overview points out, scientists keen to decipher microbial secondary metabolism face a burgeoning growth of software tools, which differ in input requirements, reconstructed attributes, and algorithmic frameworks (Table 3). Despite the inclusion of performance evaluations in most newly introduced BGC predictors, navigating these tools—whether across different categories or within the same category with varying settings—remains a substantial challenge.

The underlying causes of improved tool performance are often unclear and cannot always be ascribed to improved algorithmic architecture or training data. This underscores the need for standardized software evaluation using data sets with established biological ground truths. Such efforts would support researchers in selecting the most suitable tools and enable developers to better understand what design features contribute to successful modeling and prediction. The wealth of genetically encoded secondary metabolites suggests that any benchmarking study should take into account the intended use of the software, apply metrics that are appropriate for the question being asked—as no single evaluation metric fits all purposes—and adopt the most unbiased evaluation setting possible. We put forward that the benchmarking of tools developed to mine (meta)genomes for BGCs should be subjected to a community-wide effort in the wake of the initiative known as DREAM, standing for Dialog for Reverse Engineering Assessments and Methods (131).

Finally, we emphasize the importance of exploring options beyond the scope of our review that are particularly relevant to translational applications. Integrating sequence-based BGC prediction with complementary data types, such as transcriptomic (55), proteomic and metabolomic features (30), and structural modeling data (53), has already shown its value in providing deeper insights into the biosynthetic potential of the human microbiome and advancing natural product discovery.
